# Flipped classrooms in higher education during the COVID-19 pandemic: findings and future research recommendations

**DOI:** 10.1186/s41239-021-00316-4

**Published:** 2022-02-28

**Authors:** Blaženka Divjak, Bart Rienties, Francisco Iniesto, Petra Vondra, Mirza Žižak

**Affiliations:** 1 Faculty of Organization and Informatics, University of Zagreb, Pavlinska 2, Varaždin, Croatia; 2 Institute of Educational Technology, The Open University, Walton Hall, Milton Keynes, UK; 3 School of Medicine, University of Zagreb, Šalata 3, Zagreb, Croatia

**Keywords:** Flipped classroom, Systematic literature review, COVID-19 pandemic, Learning design, Online learning, Higher education

## Abstract

Flipped classroom (FC) approaches have gotten substantial attention in the last decade because they have a potential to stimulate student engagement as well as active and collaborative learning. The FC is generally defined as a strategy that flips the traditional education setting, i.e., the information transmission component of a traditional face-to-face lecture is moved out of class time. The FC relies on technology and is therefore suitable for online or blended learning, which were predominant forms of learning during the COVID-19 pandemic (March 2020–July 2021). In this paper we present a systematic literature review (SLR) of studies that covered online FC approaches in higher education during the pandemic. We analyzed 205 publications in total and 18 in detail. Our research questions were related to the main findings about the success of implementation of online FC and recommendations for future research. The findings indicated that those who had used FC approaches in face-to-face or blended learning environments more successfully continued to use them in online environments than those who had not used it before. The SLR opened possible questions for future research, such as the effectiveness of the FC for different courses and contexts, the cognitive and emotional aspects of student engagement, and students’ data protection. It pointed to the need to examine different aspects of online delivery of the FC more comprehensively, and with more research rigor.

## Introduction

The COVID-19 pandemic has disrupted education with an immediate impact on higher education (HE). The economic, social, political and educational crisis caused by the COVID-19 pandemic has highlighted several existing and known problems of HE (e.g., use of teacher-centered teaching and learning, lack of digital education strategy, lack of digital competences of teachers and students) that have been exacerbated due to the pandemic. According to the Organization for Economic Co-operation and Development (OECD) survey (2021, p. 6), by the end of March 2020 all responding countries had fully closed the physical campuses of their higher education institutions (HEIs) to students because of the COVID-19 pandemic crisis, and at the beginning of February 2021, in slightly over half of those responding to the survey, physical campuses of HEIs were closed, restricting access to institutions, and inevitably shifting to online learning (OECD, 2021). In many HEIs the move to online learning has been an opportunity to expand flexible learning approaches. However, while online learning generally took place through recorded lectures and online platforms, some HEIs postponed learning and teaching due to a lack of information technology (IT) infrastructure for both students and teachers (e.g., Crawford et al., 2020; United Nations, 2020).

Many HEIs have turned to online education simply by delivering materials and video conferences, without substantial investment into finding and adopting more appropriate pedagogies (Crawford et al., 2020; Rienties & Toetenel, 2016). However, by offering students a well-balanced mix of pre-recorded lectures, links to articles and reading resources, or other appropriate learning materials through an online platform, more class time could be used to help students with topics and concepts they may not understand or want to elaborate on (Council Conclusions, 2020). Thus, innovative pedagogies, digital tools, and methods to deliver quality and inclusive education in online environments have become imperative. One such innovative pedagogy approach that has received much attention is the flipped classroom (FC), in which the information transmission component of a traditional face-to-face (f2f) lecture is moved out of class time (Abeysekera & Dawson, 2015), which can engage students and support their learning in f2f, blended and online environments.

During this COVID-19 period, around the globe, we have met many students who were burdened with a large amount of online teaching, while they got by with superficial learning (Nolan et al., 2021). Therefore, it is necessary to find a way to reach those students with their very different needs and move beyond superficial learning experiences. Flipping the classroom establishes a framework that potentially ensures that students may receive a more personalized education.

### Literature review on Flipped Classroom

Although the expression "flipped classroom" has been credited to teachers Bergmann and Sams (2012), the earliest documented use of the term “flipped classroom” to describe this pedagogical method was in 1997 by Baker (Talbert, 2017), who described it in 2000 as his fully realized vision for flipped learning, using the term “classroom flip” (Baker, 2000). Lage et al. (2000) were the first to coin the flipped education approach as “the inverted classroom”. It was the first peer-reviewed journal article intended for a broad audience giving a formal framework for flipped learning (Talbert, 2017).

The FC is an active, student-centered approach that is designed to increase the quality of the period within class (Aşıksoy & Ozdamli, 2016; Nolan et al., 2021), provides opportunities for structured, active learning (Strelan et al., 2020), and encourages students to inquire and to interact with teachers, peers, employers and learning materials. Moreover, it has potential to enable teachers to cultivate critical and independent thoughts in their students, building their capacity for lifelong learning, and thus preparing future graduates for their workplace contexts (O’Flaherty et al., 2015). As a pedagogical model, the FC requires a commitment and active participation of students in learning activities both before and in the classroom, all with the contribution of IT (Aguilera-Ruiz et al., 2017; Aprianto et al., Aprianto, Ritonga, et al., 2020, Aprianto, Purwati, et al., 2020; He et al., 2016). The FC is scalable and it can be adapted to meet students’ learning needs. It can include flipping just a particular learning unit, only a part, or even the whole course.

In most cases, students use multimedia lectures through self-paced learning prior to class, while the class time is used for student-centered learning activities (O’Flaherty & Phillips, 2015). This approach is broad, allowing for variability in the implementation of activities inside and outside the classroom. Furthermore, the FC encourages students’ engagement, inquiry, and sense of autonomy and ownership of learning. It offers students the opportunity to self-regulate their learning (He et al., 2016). The key element in this approach is to encourage students to use class time to deepen their understanding and increase their competencies at using their new knowledge. Therefore, the FC is in accordance with the learning theory of Bloom’s revised taxonomy (Anderson & Krathwohl, 2001), as students first gain basic knowledge and comprehension (the lower levels of cognitive work) outside of the classroom and focus on the application, analysis, synthesis and/or evaluation (the higher forms of cognitive work) during the class time, when they have the support of their teachers and classmates (Brame, 2013).

Students’ engagement is one of the critical factors for effective teaching and this is supported by Bryson and Hand (2007), who presented that students were more likely to engage if they were supported by teachers who established personable learning environments, demanded high results, and challenged higher order thinking. Therefore, it can be pointed out that what makes a difference in the FC is not per se the diversity of learning materials, accessed via asynchronous learning, but how they are integrated into an overall approach.

A wide range of methods can be used for pre-class preparation in the FC, including pre-recorded lectures in the form of podcasts/vodcasts, screencasts, annotated notes and captured videos; the use of pre-readings, interactive videos from an online repository, which enables students to undertake their learning in a reflective and self-paced manner (O’Flaherty & Phillips, 2015). These have been shown to improve learning, specifically the lower order cognitive skills (Prunuske et al., 2012); enhance class preparation, increase classroom interactivity and improve academic performance.

In order to implement the FC, instructors need to redesign their curriculum to integrate pre-class activities into f2f classes with active learning pedagogies so that students understand the FC and are motivated to prepare for class (Tucker, 2012). Based on a scoping review on the FC (O’Flaherty & Phillips, 2015), there appears to be some misunderstandings of the required key elements for successfully flipping the classroom and building links between the pre-class and f2f sessions. Note that there is no single model for the FC, but core features include: content in advance (generally a pre-recorded lecture), educators’ awareness of students’ understanding, and higher-order learning during class time.

While there is substantial literature on FCs before COVID-19 (e.g., Cheng et al., 2019; van Alten et al., 2019), with the unexpected and rapid pivot to online and distance learning in HE, we argue that it is important to specifically review the emerging literature coming out on FCs since March 2020. Therefore, our main aim is to review the main emerging findings about the use of FC approaches during the pandemic. Therefore, in this study we will first briefly review previous literature and systematic literature reviews (SLR) on FCs and discuss the main benefits and limitations of FCs. Secondly, we will specifically review studies that fit within the time period of the pandemic and which have implemented and evaluated FCs during the pandemic.

### Previous systematic reviews and meta-analyses on Flipped Classroom

There are several relevant SLRs about flipped or inverted classrooms. A query based on (“flipped classroom” OR “inverted classroom”) AND “higher education” AND (“meta-analysis” OR “systematic review”) in the Web of Science (August 17, 2021) gave 36 results. Half of these reviews and meta-analyses dealt with the implementation of FC in specific fields like education (Turan & Akdag-Cimen, 2020), engineering (Lo & Hew, 2019), medical (Chen et al., 2018; Evans et al., 2019), mathematics (Lo et al., 2017), and nursing (Betihavas et al., 2016).

The most highly cited meta-analyses showed that FC approaches have a positive impact on student learning. For example, a meta-analysis based on 55 publications on cognitive student learning outcomes published between 2000 and 2016 (Cheng et al., 2019) found a statistically significant effect in favor of the FC instructional strategy. Similarly, a meta-analysis (van Alten et al., 2019) that included 114 studies which compared flipped and non-flipped classrooms in secondary and postsecondary education found a small positive effect of the FC on learning outcomes, but no effect was found on student satisfaction regarding the learning environment. Another meta-analysis (Shi et al., 2020) included 33 studies and showed that FC instructions can positively influence college students’ cognitive learning outcomes compared to traditional lectures.

One of the latest meta-analyses (Bredow et al., 2021) used data from 317 studies, and focused on providing evidence of the effects of flipped versus lecture-based learning on academic (learning outcomes related to foundational knowledge and higher-order thinking), intra-/interpersonal (learning outcomes related to academic/professional skills, confidence/interpersonal skills, engagement/identification and meta-cognitive skills), and satisfaction-related outcomes (course satisfaction and instructor satisfaction) in HE. It was concluded that FC interventions produced positive gains across all the three learning domains, and significant advantages of flipped over lecture-based instruction were found for seven out of eight learning outcomes.

Advantages and challenges of FC approaches were investigated by Akçayır and Akçayır (2018) based on an SLR not exclusively for HE (but 80% out of the total of 71 studies reviewed were in HE), who concluded that the FC in education yields positive academic outcomes and reported on numerous advantages of this model (e.g., enhanced learning motivation, students' positive attitudes). They emphasized that there is insufficient evidence to warrant generalization and asked for more research. Among the challenges, they mentioned that instructors should pay more attention to the quality of instructional videos, there is a need for more interaction/communication tools to help students obtain feedback/help when they are doing tasks/homework outside class, and instructors need to examine the technology availability and competency of students before implementing the flipped model.

Further, in Lundin et al. (2018), an SLR on the FC was conducted, based on all Scopus database (n = 530) references available until mid-June 2016 and it was found that the interest for the FC was growing fast, with a slight conference preference comparing to journals and a focus on HE and STEM (science, technology, engineering and math) area contributions, with the United States of America as the predominant geographical context. Studies on FCs are dominated by studies in the HE sector and are relatively local in character. They also reported that only a few reviews of the research on the FC had been conducted until 2018 (e.g., Bishop & Verlinger, 2013; O’Flaherty et al., 2015).

The attractiveness of the FC has increased due to the COVID-19 pandemic’s effect on education worldwide and the majority of HEIs switching to online teaching. However, the utilization of the FC is still not fully exploited (Hoshang et al., 2021). There are reports that indicated that, during the pandemic shift to online learning, students were more in favour of passive forms of learning such as webinars, presentations and demonstrations (Ożadowicz, 2020). To our best knowledge, there is no meta-analysis or SLR on the FC usage during the COVID-19 pandemic.

## Methodology

Our aim was to perform an SLR of studies dealing with the implementation of the FC in an online environment in HE during the time of the COVID-19 pandemic. This study is organized around the following three research questions:

- RQ1: What are the dominant research worldviews, types of research and research methods used in relevant research dealing with use of FC approaches during the pandemic?
- RQ2: What are the most important findings about the use of FC approaches during the COVID-19 pandemic?
- RQ3: What are the recommendations for future research on the FC?

In this study, we followed the recommendations on using a multi-phase process for the SLR (Mangaroska & Giannakos, 2019; Moher et al., 2009). Specifically, the SLR in this study was performed through three phases: Phase 0—*Papers extraction*, Phase 1—*Abstract review*, Phase 2—*Complete/Detailed/Full paper review*.

**Fig. 1:**
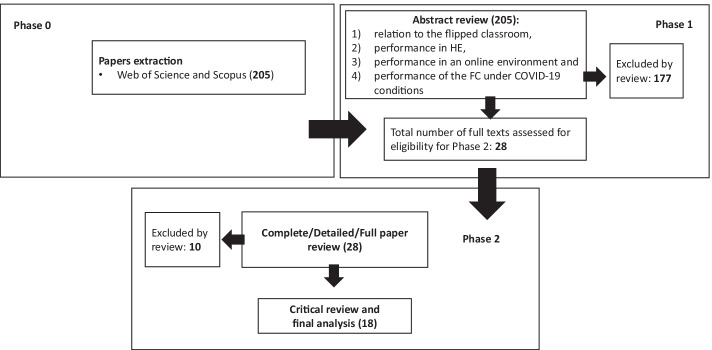
Phases included in the review

We selected two databases for searching according to the ranking as academic research databases and good coverage of studies relevant for the review—Scopus and Web of Science. The mentioned databases were searched by two researchers (BD and PV) twice—on June 2 and July 20, 2021. For the search we used the following search string: (“online” OR “remote” OR “distance”) AND (“flipped classroom” OR “inverted classroom”) AND “higher education”. Furthermore, we applied two criteria on papers: (1) published in English and (2) published between January 2020 and July 2021, which is in line with the COVID-19 pandemic. The two authors (BD and PV) manually critically screened the titles of the extracted papers to check whether they should be included in the further analysis. The defined search resulted in 205 unique publications across the two databases. The methodology of the SLR research that includes the coding process in the three phases is shown in Fig. 1.

### The coding process

The 205 extracted studies were analyzed according to defined search terms and criteria. In order to conduct the SLR in an effective and successful way, we formed an interdisciplinary team of coders, consisting of experts in medicine, mathematics, computer science, and educational technology from Croatia and the UK.

In Phase 1—*Abstract review*, identified studies were assigned for further analysis to four researchers/authors (BD, ML, MZ and PV). In this phase, the researchers reviewed and analyzed papers’ abstracts according to the following criteria: (1) relation to the FC, (2) performance in HE, (3) performance in an online environment and (4) performance of the FC under COVID-19 conditions. This analysis resulted in 28 papers which met all of the above defined criteria, while 177 studies were excluded from further analysis, mostly because they were not performed under COVID-19 conditions.

In Phase 2—*Complete/Detailed/Full paper review*, 28 papers were included, which were distributed among seven researchers/authors (BR, BD, DB, FI, ML, MZ and PV) for additional analysis. It is important to mention that the researchers did not code the same articles which they had coded in Phase 1, ensuring in that way a double check for each article. On average, each researcher coded four studies. During this phase, 28 full papers were thoroughly reviewed and for each paper the following was noted: the type of research, the most important findings, the most important recommendations for future research, the limitations of the research, the researcher’s opinion on a paper’s advantages or limitations, the country of origin, study field, sample size, the level of the analyzed course, the relation with the FC and work-based learning (WBL), and the researcher’s rating for the article based on its relevance for further analysis.

Finally, the first author (BD) critically reviewed all the double-checked papers, and based on the researchers' rates and the most important findings related to FC, selected the papers relevant for further analysis. The publications that had the lowest relevance ratings, as well those with poor methodology, were excluded. The SLR ended with 18 papers relevant for further analysis.

## Results and discussion

We analyzed 18 publications, presented in Table 1, that fulfil all the above-mentioned criteria and have findings related to the FC implementation during the COVID-19 pandemic period.

**Table 1:** Selected papers covering FC research related to the pandemic and online delivery (January 2020–July 2021)

Author(s), year	Context	Field and Level of HE	Type of research	Sample—if applicable	Main findings related to FC during the COVID-19 pandemic
Attarabeen et al., (2021)	USA students	Pharmacy undergraduate and graduate	Comparative research quantitative	192 (2018) 66 (2020)	FC was used before and during the pandemic. Student-perceived stress did not increase during online, remote learning associated with the pandemic. Additionally, datasets showed no significant differences in coping behavior, self-efficacy or emotional status
Collado-Valero et al., (2021)	Spain teachers	Education science	Comparative research quantitative	45	The results revealed an increase in the implementation of FC during the pandemic. Teachers considered that the circumstances encouraged the implementation of FC
Dapper et al., (2020)	Germany students	Medical education (radiation oncology) graduate	Case-study research mixed	125	Traditional teaching methods were largely accepted by students, but opinions on the application of alternative teaching methods or e-learning formats, in particular on the application of FC method, differed widely
Durfee et al., (2020)	USA students	Medical education (radiology) graduate	Action research mixed	111	Virtual radiology core clerkship design included online FC modules and this was shown to be successful, since student performance on the standardized final exam was similar to the in-person teaching
Feijóo et al., (2021)	Spain, Peru students	Engineering undergraduate	Action research mixed	220	Authors concluded that the flipped teaching methods implemented by them during the pandemic shift to remote teaching suffered less than other classical teaching approaches, not only with regards to lectures but also to the outcomes
Hoshang et al., (2021)	UAE students and teachers	Information systems and Engineering undergraduate	Action research mixed	300 students and 10 teachers	Students and teachers were in favor of FC usage, but not strongly and without criticism. More training in the tools and concepts of FC is required. Students in FC achieve significantly higher evaluated and assessed learning outcomes than students in traditional classrooms and are equally satisfied with the learning environment
Jia et al., 2021	China students	Education postgraduate	Community of inquiry mixed	49	Online flipped course participants performed as well as their counterparts in the conventional flipped learning format, and students’ interest levels remained constantly high throughout the online flipped classes. Analyses suggest five key factors that promote their engagement in the online flipped classes (interaction, active learning with feedback, supported problem-centric learning, teaching variation and teacher attributes)
Martinelli et al., (2021)	USA residents	Medical education (Anesthesiology) postgraduate	Non-empirical (narrative) qualitative	na	There is growing evidence that the FC is preferred by learners and may increase knowledge gain. FC works well with learning management systems to disseminate focused pre-class work
Latorre-Cosculluela et al., (2021)	Spain students	Across the university undergraduate and graduate	Action research quantitative	376	Students agreed on the benefits and effectiveness that learning with FC has on the development of twenty-first century skills for their personal and professional life. Students who took part in FC during the pandemic perceived the effects of FC on the development of competences related to “fun and learning “, “collaboration with the classmates “ and “learning from/with the classmates “ as less strong
Lee, (2020)	Korea students and teachers	Chemistry undergraduate	Non-empirical case-study qualitative	na	During the pandemic, students prefer asynchronous lectures to real-time lectures. Flipped learning was used to increase the engagement of students. Korean students asked more questions in online lectures compared to f2f
Liberman-Martin and Ogba, (2020)	USA	Chemistry undergraduate	Case-study mixed	55	Instructors found that the video-based flipped approach was readily portable to remote instruction without structural changes. During online learning students were comfortable engaging with the material, but largely uncomfortable with engaging using their microphones in the main class or breakout rooms
Liu et al., (2020)	China students	Vocational HE (digital video production) undergraduate	Case-study mixed	129	The online flipped blended teaching mode adopted in a course improves the online learning effect on students, their autonomous learning ability and problem-solving ability, and improves their interest in learning
Nepal and Rogerson, (2020)	Australia	Economics undergraduate	Semi-SLR qualitative	na	There are clear qualitative and pedagogical benefits from flipping the economics classroom, especially in introductory courses
Portela, (2020)	Portugal students	Engineering (computer science) undergraduate	Case-study quantitative	180	Students were satisfied (81%) with interactivity that combines FC with PBL, game-based learning, BYOD
Schmitz et al., (2021)	Germany students	Medical education undergraduate	Quasi-experiment quantitative	58	The integration of the WBL and FC approaches was researched and proved feasible for surgical education of undergraduates
Tang et al., (2020)	China students	Engineering undergraduate	Action research, comparative study quantitative	11,579	Students were dissatisfied with online learning in general, and especially with communication. The combined model of online teaching with flipped learning improved students’ learning, attention, and evaluation of courses. Authors claimed that in courses with difficult theories and abstract formulas, traditional teaching showed its superiority
Veldthuis et al., (2020)	Netherlands students	ICT undergraduate	Case study quantitative	na	Since the course already utilized FC before the pandemic, it was possible to give the course entirely online with minimal adjustments. Results showed that students' grades were similar to those before the pandemic and students even reported a higher level of satisfaction with the course when given remotely
Wang and Chen, (2020)	China students and teachers	Across the higher vocational colleges	Comparative study quantitative	262 teachers and 986 students	FC advantage is the flexible timing for students, and the combination of asynchronous and synchronous enhances the quality of students-teacher interaction. The difficulty is that teachers should design asynchronous and synchronous delivery carefully, prepare rich resources, plan answering time etc

### Sample analysis

**Fig. 2:**
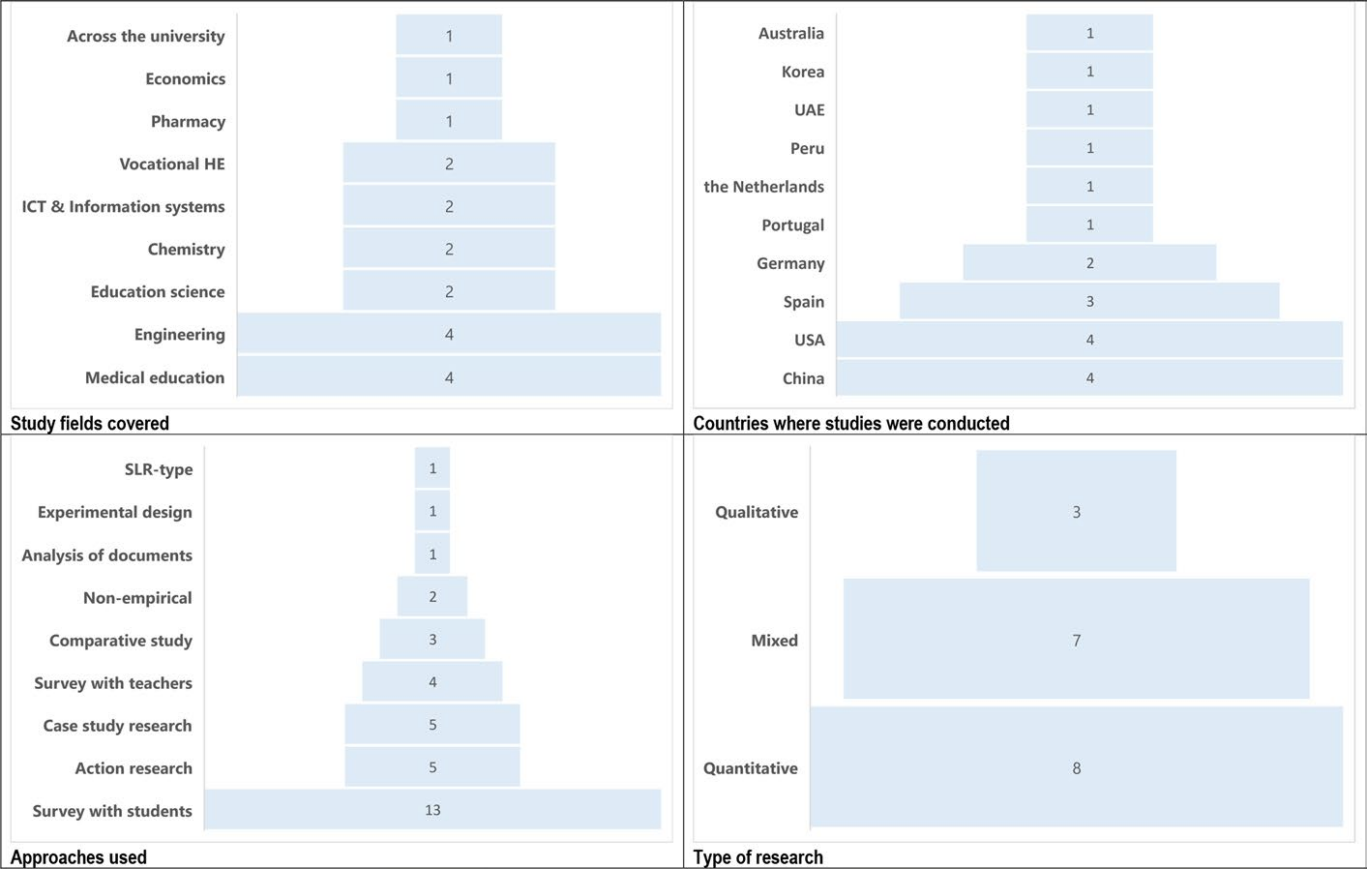
Study fields, countries, approaches and types of research covered in the sample

In the selected 18 publications, a broad variety of study fields were covered (Fig. 2), starting with Medical education (4), Engineering (4), followed by Education science (2), ICT & Information systems (2), Chemistry (2), Vocational HE (2), and finally with one study in Pharmacy, one in Economics and one across the disciplines of a university.

Four studies originated from China, four from the USA, three from Spain, two from Germany, and one was conducted in each of the following countries: the Netherlands, Portugal, Peru, United Arab Emirates, Korea and Australia. Details are given in Fig. 2.

### RQ1. Research philosophical worldviews, approaches and methods

In order to analyze the level of research rigor, the consistency of research design and the studies’ theoretical foundation, we analyzed research worldviews, types of research and methods used. Let us first briefly describe the terminology reference points and then answer RQ1.

As the authors of this paper expected, most of the studies we examined belonged to a pragmatic worldview. Instead of focusing on the methods, most studies positioned the research problem in the center of their research and tried to find methods that could help them to understand or even solve a problem (Creswell, 2014, p. 10). Obviously, in our case the challenge was how to organize effective online HE during the COVID-19 pandemic. At the same time, seeking for the understanding of the “new normal” we are living in encouraged researchers to engage with the world they were interpreting, which is mainly a constructivist worldview (Creswell, 2014, p. 8–9). Finally, many researchers are educated in the postpositivist worldview, which is predominantly deterministic, includes the scientific method, and the need to identify and assess the causes that influence the outcomes (Creswell, 2014, p. 7), and they applied their quantitative approaches and used quasi-experiments in the research of online education in the crisis caused by the COVID-19 pandemic.

We analyzed the type of research that was predominantly used in each of the selected studies. Action research [which is often used in education research, when the researcher is also a practitioner who wants to improve her/his teaching practice (Cohen et al., 2010, p. 297)], is a type of research study which is more action-oriented in order to solve an immediate problem, and is therefore closer to the pragmatic worldview. Case study research (Yin, 2014, p. 2) examines a particular contemporary event in-depth (questions “how” and “why”), but mainly by observing a problem when a researcher has little or no control over the event, not solving it like in action research.

The majority of the studies reported in Table 1 were based on a one-case study or action research of FC-related implementation of online teaching and learning during the pandemic. Most of them used questionnaire-based surveys with students (13), and a few implemented surveys with teachers. In addition, several studies used interviews with teachers or the community of inquiry. There were also examples of comparative analysis (3), document analysis, experimental design, and semi SLR research. Two studies were non-empirical, narrative studies based on observations and reflections. Details are shown in Fig. 2.

According to Creswell (2014), research design can use qualitative, quantitative or mixed methods to conduct a study. Within the group of the selected publications (Table 1), eight used quantitative methods, three used qualitative methods and seven adopted a mixed method approach. Details are shown in Fig. 2. Creswell (2014, p. 3–19) described that qualitative approaches and methods are more in line with the constructivist worldview. Quantitative designs are more inclined to post-positivist knowledge claims, and often encompass a survey and experimental research. Finally, mixed methods approaches, when a researcher collects both quantitative and qualitative data, are characteristic of pragmatic knowledge claims.

To conclude, most of the studies focused on one example of an FC and adopted a predominantly pragmatic research worldview. The results we got are comparable with Lundin et al. (2018) and Akçayır and Akçayır (2018) because both SLRs found that the research on the FC is quite scattered, mainly using case-study research focused on one course, providing local evidence of implementation.

### RQ2. The most important findings

Research (e.g., Collado-Valero et al., 2021) indicates an increase in the implementation of the FC during the pandemic because it can help maintain interactivity (Martinelli et al., 2021; Portela, 2020).

The authors of the 18 studies analyzed different aspects of implementation, but the majority (13 out of 18) took into consideration student perspectives of the quality of online teaching and learning. As expected, many difficulties were encountered at the beginning of online teaching during COVID-19. In several studies students reported to be dissatisfied with online learning in general, and especially with communication with teachers (Tang et al., 2020; Dapper et al., 2020). Furthermore, teachers noticed problems with student engagement in online learning. Analyses by Jia et al. (2021) suggested five key factors that promoted student engagement in the FC: interaction, active learning with feedback, supported problem-centric learning, teaching variation and teacher attributes.

In addition, the satisfaction of students and teachers with online FC approaches differed across disciplines and educational tradition contexts**,** but also within a discipline and institutional culture and context. For example, in medical education a one-case study incorporating the perspective of German students (Dapper et al., 2020) reported that students' perspectives differed widely. In contrast, results from a USA context showed successful implementation of FC in virtual radiology core clerkship (Durfee et al., 2020).

Several studies showed that the introduction of FC approaches overcame some of the negative aspects of the shift to remote teaching. According to Tang et al. (2020), the combined model of online teaching with the FC improved students’ learning, attention, and evaluation of courses. Another study (Liu et al., 2020) reported an improvement in the online learning effect on students, their autonomous learning ability and problem-solving ability, as well as learning. A Korean study confirmed that the FC was used to increase the engagement of students (Lee, 2020). Furthermore, research (Jia et al., 2021) indicated that online learning communities in the FC can help to create a feeling of connectedness among learners and instructors (i.e., social presence). The creation of an instant messaging chat group involving all students and teachers in the class could further promote social presence in the online FC.

Several studies (Attarabeen et al., 2021; Collado-Valero et al., 2021; Jia et al., 2021; Liberman-Martin & Ogba, 2020; Veldthuis et al., 2020) showed that in courses or study programs that had already utilized the FC before the pandemic, it was possible to give the course or a study program entirely online with minimal adjustments. Students even reported a higher level of satisfaction with the course or the study program (Veldthuis et al., 2020), and their interest remained consistently high through the online FC (Jia et al., 2021). This fitness, as the ability of an HEI or an HE system to adjust to challenges such as the pandemic, might come from the innovative profile of an HEI and its active search for better solutions, as well as systemic support for innovation. Fitness contributes to resilience. A similar result was confirmed by Svetec and Divjak (2021) in relation to national school systems that successfully reacted to the pandemic in the first semester of 2020.

The FC combined with other approaches, such as problem-based learning (PBL), game-based learning, bring your own device (BYOD) (Portela, 2020), WBL (Schmitz et al., 2021) and massive open online courses (MOOCs) (Bralić & Divjak, 2018; Jia et al., 2021) proved to be a successful combination for some study fields, like medical education or computer science.

Further recognized advantages of the FC were the flexible timing for students’ learning, the possibility to combine asynchronous with synchronous teaching and learning, and enhanced quality of student–teacher interaction (Wang & Chen, 2020). In addition, a study from Germany (Liberman-Martin & Ogba, 2020) showed that during online FC learning students were comfortable engaging with materials, but were largely uncomfortable with engaging using their microphones in the main class or breakout rooms. A further disadvantage of the online FC was that teachers had to invest a lot of time in preparation and communication. For example, to design asynchronous with synchronous stages carefully, prepare rich resources, and plan answering time required substantial workload of teachers (Wang & Chen, 2020).

Students and teachers were in general supportive of the online FC, but neither overwhelmingly or without criticism. Students expressed a need for teachers’ guidance in the learning process and teachers recognized students' need for support (Hoshang et al., 2021). Furthermore, according to Hoshang et al. (2021) and Lee (2020) students and teachers needed more training on online FC approaches. For students this also meant training in self-regulated learning, time management and commitment to education.

A certain proportion of students were against the online FC (about 12% in Hoshang et al. (2021) were strongly against) and the reasons for that need to be researched further. For example, a study performed in Germany (Liberman-Martin & Ogba, 2020) showed that students were not comfortable with some ways of direct synchronous communication, and a study from the Korean context also reported that during the pandemic students preferred asynchronous lectures to real-time lectures (Lee, 2020). At the same time, the Korean study (Lee, 2020) reported that Korean students asked more questions in online lectures compared to f2f. In general, results from Akçayır and Akçayır (2018) on how to improve the online FC delivery can be used (the quality of instructional videos, more interaction tools to help students to obtain feedback outside the class, technology availability and competency of students before implementing the flipped model).

A further topic that was covered in these 18 studies was student assessment results during the pandemic in comparison to the pre-pandemic situation. Studies that incorporated the use of the online FC during the pandemic (Jia et al., 2021; Veldthuis et al., 2020) reported no significant difference in students' learning outcomes during the pandemic compared to conventional FC performed before the outbreak of the pandemic.

Finally, the FC has been recognized (Latorre-Cosculluela et al., 2021; O’Flaherty & Phillips, 2015) as the teaching and learning approach that contributes to the development of twenty-first century skills. Competencies also include character building, collaboration, communication, citizenship, critical thinking and creativity. However, the studies included in this review indicated that students who participated in the online delivery perceived peer-learning, collaborative learning and the joy of learning as less experienced. There were also some issues related to online FC delivery that provoked uneasiness and stress among students (Attarabeen et al., 2021). Experts emphasized that the shift to the online delivery of HE was very stressful for students and teachers. But if online delivery was prepared with pedagogical care, it can be less stressful. For example, Attarabeen et al. (2021) stated that student-perceived stress did not increase during online, remote learning associated with the pandemic. Additionally, the study showed no significant differences in coping behavior, self-efficacy or emotional status.

One of interesting findings (Feijóo et al., 2021) was that almost a half of the surveyed students lacked the fundamentals needed to properly acquire the subject skills and learning (previously mathematics was mentioned), and the drop-out rate in fundamental subjects was notably higher than in technological modules. Furthermore, in Hoshang et al. (2021) teachers expressed their opinion that the online FC would be more suitable for a theory-based lesson, and that it would be challenging for the practical lab part of lesson sessions. In contrast, Tang et al. (2020) reported on a survey on more than 11,000 engineering students in China and claimed that in courses with difficult theories and abstract formulas, traditional teaching showed its superiority in comparison to FC integrated online learning. Additionally, Nepal and Rogerson (2021) suggested that there were pedagogical benefits from flipping the economics classroom, especially in introductory courses in economics, which are often theoretical and quantitative. Finally, the OECD’s findings (2021) also supported the opinion that online teaching in general is more suitable for postgraduate study programs and less suited to subjects with a strong practical component (nursing, medicine, natural sciences). Obviously, our studies seem to be inconclusive, even contradictory, and further research is needed.

**Fig. 3:**
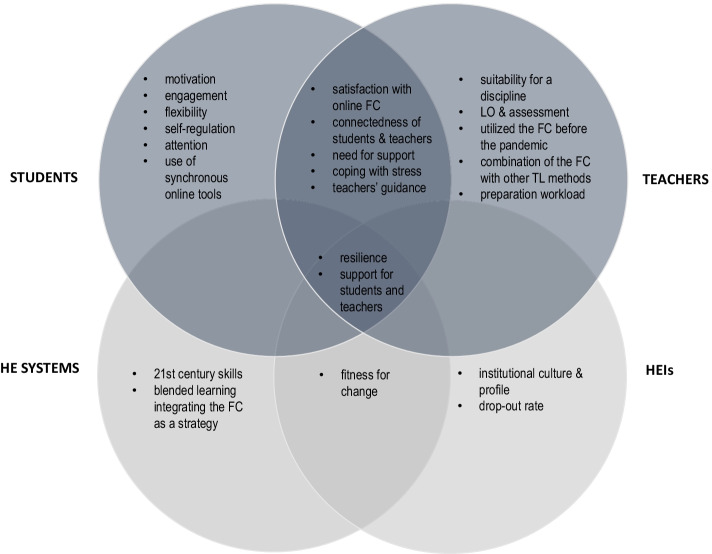
An overview of the major topics explored by the studies

Despite the aforementioned concerns, these 18 studies contain useful recommendations and good practices for other HEIs (e.g., Wang & Chen, 2020). Martinelli et al. (2021) concluded that we must seek new and innovative solutions to age-old problems in education, rather than waiting for things to “return to normal”. Furthermore, some authors (e.g., Schmitz et al., 2021) found grounds for establishing blended learning concepts integrating the FC in the future of education. A thematic overview of the major topics explored in the included studies is presented in Fig. 3.

### Limitations of the analyzed studies

The limitations of the SLR are related to the majority of the publications being restricted to one-case study research in an institution or one study field, and often the samples were not representative. Nevertheless, all these studies were of practical value and many HE teachers and decision-makers were interested in them. Therefore, it was important these studies were published as soon as possible, even though some of them were not necessarily based on a sound research methodology, and did not involve any experiments. Additionally, student and teacher perspectives can be biased because they might not have had experiences with the online FC before the pandemic, or had a scarce encounter during the pandemic. For example, Dapper et al. (2020) surveyed 125 German medical students whose acceptance of e-learning methods was not very high and the authors stated that it was possible that some students had no experience with the FC and therefore could not evaluate its potential benefits. Furthermore, the use of the final grade as a single proxy of achievement when analyzing students’ academic performance in emergency remote teaching (e.g., Iglesias-Pradas, 2021) can be a limitation. Therefore, it is crucial to enhance the forms, methods and means of monitoring and evaluation of students’ achievements in the online FC (Shtaleva et al., 2021). In a two-context study (Feijóo, 2021) it is not clear what authors learned from Spain's situation compared to Peru or what are the differences between the perspectives of students on undergraduate and graduate level. It should also be noted that the SLR included only studies which were written in English, which is due to the international composition of the research team, with English language being the only common language. All these limitations in the methodology cause the limitations of this SLR-research. Obviously, this SLR-research covers only the first 18 months of the COVID-19 disruption to education and there is probably more research emerging over time. But for practitioners and researchers our research can be a valuable reference point.

To conclude, among the findings that are in line with the previous research are those that the FC can be a useful approach for engaging students with learning materials as well as with their peers and teachers. But at the same time, not all students were happy with the innovative online FC approach, for example, when it came to synchronous two-way modes of communication. Furthermore, FC approaches are more demanding for teachers, especially in relation to the time needed for preparation. Therefore, there might be a need for more institutional support for meaningful online delivery as well as training for teachers and students, which is in accordance with Akçayır and Akçayır (2018).

An interesting result is that the combination of the FC with other innovative and relevant approaches to teaching and learning (such as WBL, PBL, the use of MOOCs, game-based learning) can have a higher impact on student learning and satisfaction with it. Therefore, it can be recommended to use a holistic learning design that aligns teaching and learning strategies and approaches to learning outcomes and use a combination of them that is best suited to the intended learning outcomes as well as the needs and characteristics of the student body. Step by step changes, monitoring and evaluation of the implementation of innovative strategies and approaches are healthy for the fitness of the system, which is also confirmed as a very positive element for the resilience of the educational system to extreme and rapid disruptions. Namely, those who had used FC approaches in f2f or blended learning environments, more successfully continued to use them in online environments.

### RQ3. Recommendations for future research

Our third and final aim of this SLR (RQ3) was to give some recommendations for future research about the implementation of the online FC. Therefore, we investigated specifically the selected 18 publications listed in Table 1 based on authors’ explicit recommendations for future research, but we (authors of this paper) also identified some research gaps based on a comparative analysis of the broader set of studies identified in the SLR.

Factors that can support the resilience in the emergency situation need to be investigated in general (Attarabeen et al., 2021), and more statistics and metrics on student learning and performance comparing on-campus with online FC across different fields of teaching and learning are needed (Latchman et al., 2021). It seems that the FC works well during the pandemic if it has already been introduced before the pandemic in some form, and it would be interesting to research further if the transition to online teaching was “easier” for those that had used the FC or other innovative teaching and learning approaches before.

Latorre-Cosculluela et al. (2021) pointed out that future research should investigate the links between students’ perception of usefulness of the approach, the simplicity of its use, and the acceptance of its integration in teaching and learning at their university. They also pointed out that further studies are needed to compare the FC with non-inverted models, to explore how effective this approach actually is.

There is no one FC approach. Indeed, as is evident from this SLR, there were diverse ideas and practices regarding the application of the FC with e-learning methods, which makes it difficult to implement (Dapper et al., 2020). Therefore, a systematic research of literature and practices on types of online FC might inform practitioners and HE decision-makers. Further research on combining the FC with WBL, PBL, game-based learning and MOOCs also seems promising.

Portela (2020) and Schmitz et al. (2021) as well as the OECD (2021) stressed the importance of the confidentiality of learners’ personal information and data protection issues (GDPR rules), because it is more difficult to protect all critical data in an online environment. Nepal and Rogerson (2020) recommended that future research in economics education should focus more on capturing the cognitive and emotional aspects of student engagement to inform policy-making in promoting student engagement. Future research should, according to them, focus on online classrooms, such as MOOCs, and the comparative effectiveness of hybrid forms of subject delivery.

Jia et al. (2021) indicated that online learning communities can support engagement and should be researched further. Whereas Lee (2020) and Liberman-Martin and Ogba (2020) indicated that students hesitated to engage in two-way synchronous communication using voice or video, in another study (Jia et al., 2021) students preferred when instructors required using webcams over the classes in which using webcams was optional. This might imply some differences in culture and educational traditions and a possibility to increase student activity in an online environment compared to f2f based on that research. Attarabeen et al. (2021) proposed an impressive list of recommendations for future research on stress of students. They argued that future research could also explore faculty-perceived stress during the COVID-19 pandemic and the subsequent move to online learning. Liberman-Martin and Ogba (2020) mentioned assessment issues with cheating, time limitations and an open-book approach as an overall concern of online assessment. Indeed, assessment in the online FC is still an open research question, especially when combined with the reliability concerns about peer-assessment (Divjak & Maretić, 2017).

Based on comparing the results of the selected studies, we propose the following research questions. We propose further research regarding the effectiveness of the FC for different subjects/courses/study programs. Are the FC and other approaches using either web-based technology or f2f teaching equally effective in fundamental courses compared to technological/practical ones? Are FC approaches more suitable for theory-based subjects (such as mathematics) or for practically-oriented courses? Is the FC equally useful for undergraduate and graduate subjects/courses? There were subject-oriented studies before the pandemic, but it is difficult to compare them with contemporary studies because the previous ones were not related to online delivery. Furthermore, what ways of combining the FC with WBL, PBL, game-based learning and MOOCs may be effective?

Moreover, it would be useful to investigate the FC in different contexts. What are the cultural educational characteristics and traditions to take into consideration when implementing the FC? Are there cultural differences in the acceptance of student-centered learning as well as in the students’ willingness to learn without strong teacher guidance and authority? What are the links between students' perception of FC approaches and the ways these are integrated into teaching and learning at their HEIs?

Furthermore, Martinelli et al. (2021) advocated for the development of novel approaches, adaptation of new tools based on the best evidence and educational theories, with a focus on interactivity without physical proximity and balancing costs and benefits. In line with that, it would be worth exploring what evidence we need, how we can provide it and use it for the development and implementation of novel approaches.

Following the above-mentioned research, it would be useful to further investigate students' engagement. In what ways can students' engagement in the FC be promoted? What cognitive and emotional aspects should be taken into account in this respect? In what ways can online learning communities support students' engagement?

It is also important to gain a deeper insight in the stress related to online teaching and learning, including the online FC. In comparison to other models of learning and teaching, what is the impact of the online FC on students and the faculty in terms of stress? What educational strategies may lead to the least stressful classroom experiences?

Further research on the assessment in the online FC is essential. How can the reliability and validity of online assessment aligned with FC approaches be ensured? What forms of assessment and institutional action on academic integrity can have a positive impact on preventing cheating?

Finally, in general it is essential to elaborate in more detail the research strategies that include philosophical worldviews, theoretical foundations, research approaches, research design and research methods (Creswell, 2014, p. 5) and apply more research rigor to validate research instruments and use larger samples, introduce new variables, replicate studies in other universities and countries and compare results (Attarabeen et al., 2021; Collado-Valero et al., 2021).

To conclude, similarly as Lundin et al. (2018), we can report on a lack of systematic research on the implementation of the FC during the COVID-19 pandemic, which can be justified with the short time available to publish results related to the contemporary processes. Therefore, it is imperative for future research to examine different aspects of the online delivery of the FC more fully and with more research rigor, as well as to examine the use of the FC in different contexts. Additionally, recommendations for meaningful delivery need to be holistic to cover teachers’ and students’ opinions and needs as well as institutional strategies for innovative teaching and learning in the digital era that cover the requests for twenty-first century skills. It means that FC approaches in teaching and learning need to be studied as integrated with other teaching and learning approaches and institutional strategies.

## Conclusion

In this systematic literature analysis (SLR) about the use of online FC approaches during the pandemic 205 publications were analyzed, out of which the most relevant 18 in detail. As regards the research methods, approaches and worldviews, the majority of the studies covered only a single case of online FC implementation during the pandemic (RQ1). Many publications shared the teaching and learning practice describing how remote teaching and learning were organized when the rapid shift to online teaching and learning was made. Among them, the vast majority used quantitative or mixed method approaches to research and took the pragmatic research worldview.

In terms of the most important findings (RQ2), our SLR indicated an increase in the implementation of the FC during the pandemic, as teachers considered the online FC to help maintain interactivity. Researchers were not unanimously positive about the online FC implementation during the COVID-19 pandemic, but several studies showed that the introduction of FC approaches overcame some of the negative aspects of the shift to remote teaching. The studies showed many advantages, but also some disadvantages of online FC implementation. Among others, advantages were related to the flexible timing for students’ learning, the possibility to combine asynchronous with synchronous teaching and learning, the development of twenty-first century skills and enhancing the quality of student–teacher interactions.

Some of the disadvantages referred to teachers having to invest much time in the preparation and implementation of the online FC as well as the preparation of students, because a certain proportion of students were against FC. Studies reported no significant changes in students' achievement of learning outcomes during the pandemic in comparison with pre-pandemic situations. An interesting result was that the combination of the online FC with other innovative and relevant approaches to teaching and learning seemed to have a positive impact on students’ learning and satisfaction levels. The studies implied that teachers who had used FC approaches before the pandemic in f2f or blended learning environment were more successful in the implementation of FC in online environments during the pandemic than those who started with the implementation of the online FC when the pandemic started. This supports the general observation that educational systems that are constantly changing and trying to find innovative and meaningful solutions are more fit, and therefore resilient, to face up to disruptions like the pandemic. Nonetheless, there are several limitations of the analyzed studies, including the majority of publications being related to one-case study research and samples often not being representative.

In line with RQ3, future research might cover research questions regarding, for example, the effectiveness of FC for different subjects/courses/study programs, the implementation of the FC in different contexts, the cognitive and emotional aspects of student engagement, finding evidence to support the meaningful development and implementation of novel approaches, as well as students’ data protection. Finally, it is required for future research to examine different aspects of online delivery of the FC more comprehensively and with more research rigor.

## Data Availability

Data sharing is not applicable to this article as no datasets were generated or analyzed during the current study.

## Abbreviations

BYOD: Bring your own device
FC: Flipped classroom
f2f: Face-to-face
HE: Higher education
HEI: Higher education institution
IT: Information technology
MOOC: Massive open online course
OECD: Organization for Economic Co-operation and Development
PBL: Problem-based learning
SLR: Systematic literature review
WBL: Work-based learning
